# *Bacillus velezensis* SYL-3 suppresses *Alternaria alternata* and tobacco mosaic virus infecting *Nicotiana tabacum* by regulating the phyllosphere microbial community

**DOI:** 10.3389/fmicb.2022.840318

**Published:** 2022-07-28

**Authors:** He Liu, Jun Jiang, Mengnan An, Bin Li, Yunbo Xie, Chuantao Xu, Lianqiang Jiang, Fangfang Yan, Zhiping Wang, Yuanhua Wu

**Affiliations:** ^1^Liaoning Key Laboratory of Plant Pathology, College of Plant Protection, Shenyang Agricultural University, Shenyang, China; ^2^Sichuan Province Tobacco Company, Chengdu, China; ^3^Sichuan Province Tobacco Company, Luzhou, China; ^4^Sichuan Province Tobacco Company, Xichang, China; ^5^Sichuan Province Tobacco Company, Panzhihua, China

**Keywords:** *Bacillus velezensis* SYL-3, *Alternaria alternata*, tobacco mosaic virus, *Nicotiana tabacum*, phyllosphere microbial community

## Abstract

The occurrence of plant diseases is closely associated with the imbalance of plant tissue microecological environment. The regulation of the phyllosphere microbial communities has become a new and alternative approach to the biological control of foliar diseases. In this study, *Bacillus velezensis* SYL-3 isolated from Luzhou exhibited an effective inhibitory effect against *Alternaria alternata* and tobacco mosaic virus (TMV). The analysis of phyllosphere microbiome by PacBio sequencing indicated that SYL-3 treatment significantly altered fungal and bacterial communities on the leaves of *Nicotiana tabacum* plants and reduced the disease index caused by *A. alternata* and TMV. Specifically, the abundance of *P. seudomo*, *Sphingomonas*, *Massilia*, and *Cladosporium* in the SYL-3 treatment group increased by 19.00, 9.49, 3.34, and 12.29%, respectively, while the abundances of *Pantoea*, *Enterobacter*, *Sampaiozyma*, and *Rachicladosporium* were reduced. Moreover, the abundance of beneficial bacteria, such as *Pseudomonas* and *Sphingomonas*, was negatively correlated with the disease indexes of *A. alternata* and TMV. The PICRUSt data also predicted the composition of functional genes, with significant differences being apparent between SYL-3 and the control treatment group. Further functional analysis of the microbiome also showed that SYL-3 may induce host disease resistance by motivating host defense-related pathways. These results collectively indicate that SYL-3 may suppress disease progression caused by *A. alternata* or TMV by improving the microbial community composition on tobacco leaves.

## Introduction

Microorganisms are ubiquitous in nature and influence everyday life in various harmful as well as beneficial ways (Morelli and Pellegrino, 2021). Currently, biological control using microorganisms and their metabolites has been used as a potentially effective, sustainable, and alternative method (Zhang N. et al., 2017). The microorganisms such as the genera *Bacillus*, *Pseudomonas*, *Trichoderma*, as well as *Streptomyces* have been reported to effectively inhibit the occurrence and pathogenesis of plant diseases caused by various pathogenic fungi, bacteria, and viruses (Ashrafi et al., 2021; Gao et al., 2021; Jiang et al., 2021; Sánchez-Montesinos et al., 2021). The major *Bacillus* spp. that has been applied in biological control are *B. subtilis*, *B. megaterium*, *B. amyloliquefaciens*, and *B. velezensis* (Wang Y. et al., 2020; Zeng et al., 2021). *Bacillus* is a well-known producer of a wide array of antagonistic compounds with different structures, namely, peptides, lipopeptides, bacteriocins, and polyketide compounds (Fira et al., 2018). For instance, the *B. amyloliquefaciens* PPL strain can suppress the infection of cucumber mosaic virus as well as upregulate plant defense-related genes by producing fengycin (Kang et al., 2021). *B. velezensis* AR1 has been reported to resist *Alternaria* leaf spots caused by *Alternaria sesami* and improve plant physiological traits by inducing resistance in the host (Bayisa, 2020). Bacillomycin D and fengycin produced by *B. amyloliquefaciens* LYZ69 can effectively inhibit anthracnose of *Medicago sativa* (Hu et al., 2021).

In addition to the beneficial rhizosphere microorganisms that have been well investigated in their roles in the management of plant diseases (Wang et al., 2021; Zhuang et al., 2021), some lines of research indicate that phyllosphere microbial communities, namely, those of bacteria, fungi, and protozoa are also closely related to plant health as well as disease progression (Gu et al., 2010; Yang et al., 2017). The next-generation sequencing techniques are extensively applied for comprehensive analysis of the structure of microbial communities such as phyllosphere microbiome (Zhou et al., 2018; Vu et al., 2019). The sequences of 16S ribosomal RNA or the internal transcribed spacer (ITS) are currently used for amplification to analyze phyllosphere bacterial or fungal communities, respectively, (Vorholt, 2012). Recent studies indicated that the application of beneficial microorganisms, as well as microbial pesticides, can regulate the microbial community composition of the phyllosphere, thereafter promoting plant fitness and alleviating symptoms (Vorholt, 2012; Podolich et al., 2015). For example, foliar application of *B. megaterium* PB50 improved plant growth under various stress conditions by changing the structure of phyllosphere microbiota during the reproductive stage of rice (Arun et al., 2020). *B. amyloliquefaciens* B1408 was reported to promote plant growth and suppress cucumber fusarium wilt disease by changing the microbial community composition in the rhizosphere (Han et al., 2018). Studies also showed that *Pseudomonas putida* KT2440 can drastically reduce necrosis on the leaves of *Nicotiana benthamiana* by influencing the microbial composition of the rhizosphere and phyllosphere (Bernal et al., 2017).

The yield and quality of crops are affected by many factors, among which the occurrence of diseases is usually the dominant one (Pan et al., 2021). *Alternaria alternata* is a facultative saprophytic fungus infecting over 400 species of host plants and ubiquitously causes leaf spot or leaf blight by synthesizing various kinds of mycotoxin, which pose threats to food safety and human health (Liu et al., 2020; Huang et al., 2021). Epidemiological studies also demonstrated that *A. alternata* released a large number of fungal spores in the atmosphere, making the disease hard to prevent and control (Woudenberg et al., 2015). Tobacco mosaic virus (TMV) is also a well-investigated plant virus with broad host ranges and causes significant economic losses in agriculture (Roossinck, 2015). The two pathogens above are ubiquitous and serious, causing huge losses to crop yield and agricultural economy every year (Wang et al., 2017; Chen et al., 2020). Traditional management methods, such as seedling grafting, crop rotation, and chemical strategies, have been suggested to control tobacco diseases, but these approaches are not economical, reliable, or environmentally friendly (Zhang et al., 2020).

Compared with in-depth research on the role of root colonization microbiota in plant health (Palmieri et al., 2016; Shi et al., 2016, 2017), few studies have been conducted on the potential interactions between phyllosphere microbial communities and plant diseases. In this study, the newly identified *B. velezensis* SYL-3 showed a significant inhibitory effect on *A. alternata* and TMV. In addition, a microbiome high-throughput sequencing was performed and the results suggested that SYL-3 can reduce the incidence of tobacco leaf diseases probably by promoting the phyllosphere microbial community structure, which provided a valuable theoretical basis for the biological control of tobacco diseases.

## Materials and methods

### Biomaterials and growth conditions

Biocontrol strain SYL-3 and *A. alternata* were isolated from tobacco leaves in Luzhou (105°26′E, 28°52′N), Sichuan Province of China, which were isolated and stored on the potato dextrose agar (PDA) and Luria–Bertani (LB) medium (Solarbio, Beijing, China) at 4°C, respectively, and were activated at 28°C for subsequent experiments. TMV virions were purified from pCB-TMV-SY (no. MG516107) inoculated *N. benthamiana* leaves according to Gooding’s method and diluted to 20 μg/mL with 10 mM phosphate-buffered saline. The *Nicotiana glutinosa* and *Nicotiana tabacum* cv. NC89 plants were cultivated at the 26°C growth chamber and were treated at the fifth to seventh leaf stage for subsequent experiments.

### Bacterial identification

The strain SYL-3 was identified by Gram staining, morphology, physiological, and biochemical tests according to Bergey’s Manual of Determinative Bacteriology (Brenner et al., 2005). Further identification of SYL-3 was confirmed by the analysis of 16S rRNA and *gyrA* gene sequences. Genomic DNA was isolated according to the standard phenol:chloroform procedure. The 16S rRNA gene was amplified by PCR with the bacterial universal primer pair 27F:5′-AGTTTGATCMTGGCTCAG-3′ and 1492R:5′-GGTTACCTTGTTACGACTT-3′ (Marchesi et al., 1998). Primer pair *gyrA*-F:5′-CAGTCAGGAAATGCGTACGTCCTT-3′ and *gyrA*-R:5′-CAAGGTAATGCTCCAGGCATTGCT-3′ was used to amplify the partial gene sequence of *gyrA* (Yang et al., 2017; Zhang X. et al., 2017; Dhruw et al., 2020; Wu et al., 2021). The obtained PCR products were sequenced (Sangon Biotech Co., Ltd) and BLAST alignment was performed in GenBank according to sequence homology. Then, phylogenetic analysis was performed using MEGA7.0 by the neighbor-joining method with 1,000 bootstrap replications.

### The colonization of the SYL-3 strain

*Bacillus* competent cell preparation and electrotransformation were performed according to a previous study (Wang B. et al., 2020). SYL-3 was transformed with a pGFP78 plasmid that was kindly provided by Prof. Wang Qi from China Agricultural University. The SYL-3 expressing GFP was designated as SYL-3-*gfp* and incubated in a 100 mL of LB agar medium (containing 50 μg/mL of ampicillin and tetracycline) at 28°C, 180 r/min. Then, *N. tabacum* cv. NC89 plants were treated with SYL-3-*gfp* bacterial suspension (10^9^ CFU/mL), and the colonization of SYL-3-*gfp* strain on tobacco leaves was detected based on fluorescence signal. The GFP fluorescence was photographed under ultraviolet illumination by a B-100AP longwave-UV lamp (Ultra-Violet Products, Upland, CA, United States.) and laser scanning confocal microscope (Olympus Co., Ltd, Japan, FV3000).

### Effects of SYL-3 on pathogenic microorganisms

The inhibitory effect of SYL-3 against *A. alternata* was determined using *in vitro* antagonism assay as previously described (Ren et al., 2012). Briefly, a fungus plug measuring 5 mm in diameter was placed at the center of the PDA, and 1 μL of bacterial culture (10^9^ CFU/mL) was deposited on both sides of the fungus block. All tests were performed in triplicate. The plates were incubated at 28°C for 4 days, at which time the antifungal eff icacy of treatments was evaluated and recorded. Leaves of *N. glutinosa* at the same position were inoculated with 100 μg TMV virions and treated with SYL-3 bacterial suspension (10^9^ CFU/mL) or ningnanmycin (An et al., 2019) at 12 h post-inoculation. Meanwhile, leaves inoculated with TMV virions (100 μg) and treated with sterile water only were served as control treatments. The local lesion numbers were recorded 3–4 days post-inoculation.

### Plot test

Plot experiments with four treatments, namely, SYL-3 bacterial suspension, ningnanmycin (8% water, Deqiang Biology Co., Ltd., China), dimethachlon (40% wettable powder, Jiangxi Heyi Chemical Co., Ltd., China), and sterile water were conducted in Luzhou planting field (105°26′E, 28°52′N) using tobacco variety Yunyan No. 87. Those two pesticide treatments were served as positive control groups and water treatment was used as blank control. Each agent treated 60 tobacco plants per treatment and consisted of three biologically independent replicates. The concentration of the bacterial suspension is 10^9^ CFU/mL, and dimethachlon and ningnanmycin were diluted to 36 μg/mL and 75 μg/mL in the field experiment, respectively. Each plant was treated with about 30 mL of bacterial suspension and the leaves were sprayed evenly on both sides, at 0, 7, and 14 days, respectively.

### Disease incidence estimating, sample collection, and phyllosphere microbe elution

A random sampling method was used to select five points in each plot, and four tobacco plants were selected at each point and marked to investigate the disease index on 16 June 2021 (before SYL-3 application), 23 June 2021 (7 days after SYL-3 application), 30 June 2021 (14 days after SYL-3 application), and 7 July 2021 (21 days after SYL-3 application), respectively. The degrees of disease infection (*r*) were classified into six grades (0, 1, 3, 5, 7, and 9) as previously described (Sama et al., 2019). The mean disease index of diseased tobacco plants was calculated by the following equation:


Diseaseindex(%)=[∑(r×ni)/(nt×R)]×100

where *r* represents the degree of disease infection, *ni* represents the number of infected plants corresponding to the grade of *r*, *nt* represents the total number of tested plants, and *R* represents the value of the highest degree of disease infection among the plants. The disease control rate was calculated according to the following equation:


Diseasecontrolrate(%)=(Ci-Ti)/Ci×100

*Ci* represents the disease index of control and *Ti* represents the disease index of the treatments. After the fourth disease investigation, five fields were selected by random sampling method in SYL-3 bacterial suspension and blank control-treated plots. Twenty randomly selected tobacco plants and four leaves per plant were collected from each treatment group. Thereafter, 10 g of each sample was vibrated in 100 mL of sterile water at 170 r/min for 30 min and then under ultrasonic conditions for 5 min. The vibration and ultrasonic operations were repeated three times. Finally, the supernatant was transferred to 50 mL centrifuge tubes, centrifuged at 12,000 r/min for 10 min to collect the precipitated microorganisms, and quickly placed in liquid nitrogen for subsequent experiments.

### DNA extraction, amplification, and sequencing of phyllosphere microbe

Total DNA of phyllosphere microbial samples was extracted from samples using the Microbial DNA Extraction Kit (UltraClean^®^ Microbial DNA Kit, MoBio, United States) according to the instructions. Using the extracted DNA as a template for PCR reaction, the V3–V4 region of the bacterial 16S rRNA gene and the ITS1 region of the fungal 18S rRNA gene were amplified with respective primer pairs combined with adapter sequences and barcode sequences (The bacterial 16S rRNA primer sequences: Forward primer, 5′-AGTTTGATCMTGGCTCAG-3′; reverse primer, 5′-GGTTACCTTGTTACGACTT-3′; the fungi ITS1 primer sequences: Forward primer, 5′-CTTGGTCATTTAGAGGAAGTAA-3′; reverse primer, 5′-GCTGCGTTCTTCATCGATGC-3′). The Quant-iT™ dsDNA HS reagent (Thermo Fisher Scientific, Waltham, MA, United States) was used to quantify and pool all PCR products. High-throughput sequencing was carried out on the PacBio sequencing platform (Biomarker Technologies Co. Ltd, China). The SMRT Cell method (Jones and Kustka, 2017) was used to sequence marker genes, and then circular consensus sequencing (CCS) was filtered by UCHIME v4.2^1^ software to acquire Optimization-CCS. The sequence was clustered by operational taxonomic units (USEARCH v10.0)^2^ at a 97% similarity level, then the species composition of samples can be revealed by species annotation and richness analysis (RDP Classifier v2.2)^3^. Alpha diversity, Beta diversity, and significant species difference analysis were carried out by MOTHUR (version v1.30)^4^ to discover the differences between samples. The raw reads generated by PacBio sequencing were submitted to the Sequence Read Archive database at NCBI (SRA)^5^, with the SRA BioProject accession number [PRJNA790673 (Bacterial communities 16s rRNA sequencing results)] and [PRJNA790671 (Fungal communities ITS sequencing results)].

### Functional gene prediction

The PICRUSt software was used to infer the functional gene composition in the samples by comparing the species composition information obtained from the 16S sequencing data, thus analyzing the functional differences between different samples or subgroups (Parks et al., 2014). The differences and changes in the metabolic pathways of microbial community functional genes among samples of different groups were predicted through the difference analysis of the KEGG metabolic pathways (Wilkinson et al., 2017).

### Statistical analysis

All data and Spearman correlation analysis were analyzed using the SPSS 20.0 program (SPSS Inc., Chicago, IL, United States), and the one-way analysis of variance with Duncan’s test was used to assign significance as *P* < 0.05, using SparCC algorithm for correlation analysis. The correlation between the sample and other species at a certain taxonomic level can be achieved through Pearson’s correlation network analysis. The network diagram of species correlation was generated by Python (Maucher et al., 2011). A linear discriminant analysis and effect size (LEfSe) was performed to identify microbial communities’ biomarkers with statistical differences between different treatments (Segata et al., 2011).

## Results

### Identification and colonization of biocontrol strain SYL-3

**FIGURE 1:**
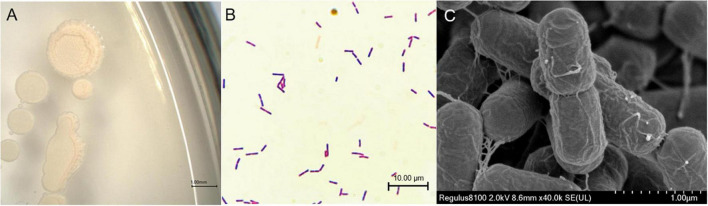
Morphological identification of SYL-3. **(A)** Morphology observation of SYL-3 on LB agar medium. **(B)** Observation of the strain SYL-3 after Gram staining. **(C)** Scanning electron micrographs of the strain SYL-3.

**FIGURE 2:**
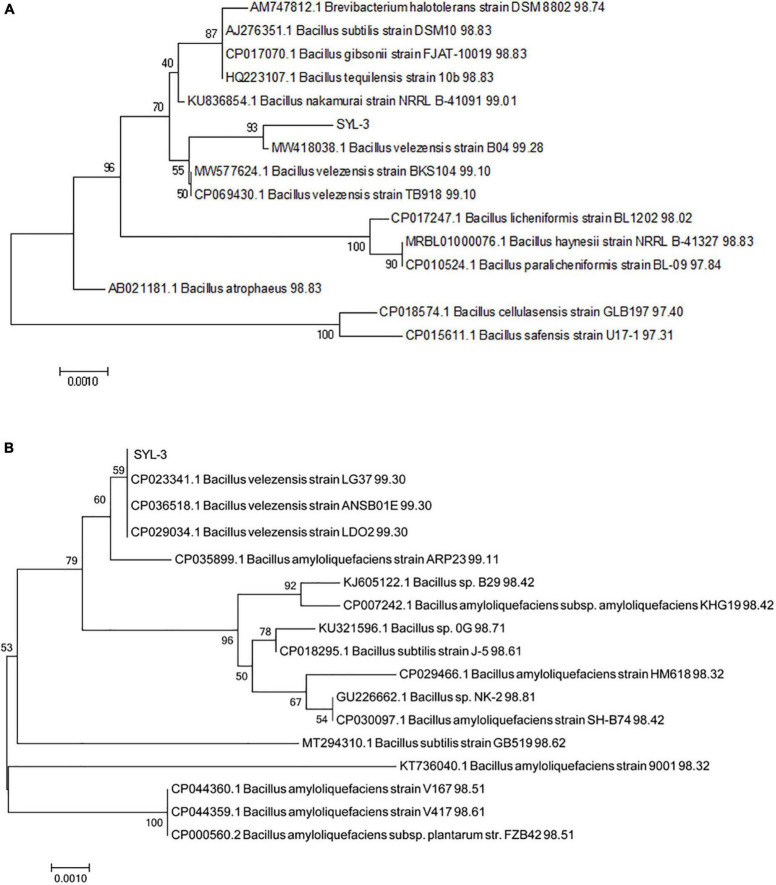
Phylogenetic analysis of strain SYL-3 based on the sequences of 16s rRNA **(A)** and *gyrA* **(B)**. The tree was constructed using the neighbor-joining algorithm with 1,000 bootstrap replications.

The strain SYL-3 was identified by morphological, physiological, and biochemical tests, along with genetic homology analysis. The results showed that the colony of SYL-3 was milky white, dim, and opaque, with a bulged center and wrinkled edges (Figure 1A). SYL-3 was Gram-positive (Figure 1B) and rod-shaped (Figure 1C). Based on the physiological and biochemical characteristics (Table 1), SYL-3 was preliminarily identified as *Bacillus* sp. Sequence alignments and phylogenetic analysis were conducted using sequences of 16S rRNA and *gyrA* gene of SYL-3. The results indicated that the 16S rRNA gene of SYL-3 was most closely related to *B. velezensis* B04 (MW418038.1), *B. velezensis* BKS104 (MW577624.1), and *B. velezensis* TB918 (CP069430.1) with 99.28, 99.10, and 99.10% sequence identity, respectively, and showed comparatively lower sequence similarity with *Bacillus safensis* U17 (CP015611.1) and *B. cellulasensis* GLB197 (CP018574.1; Figure 2A). In addition, sequencing of *gyrA* of SYL-3 showed high sequence similarity with *B. velezensis* LG37 (CP023341.1), *B. velezensis* ANSB01E (CP036518.1), and *B. velezensis* LDO2 (CP029034.1), with 99.30% sequence identity, respectively, while distantly related to *B. amyloliquefaciens* strain HM618 (CP029466.1) and *B. amyloliquefaciens* strain 9001 (KT736040.1; Figure 2B). Taken altogether, strain SYL-3 was identified as a strain of *B. velezensis*.

**TABLE 1:** Physiological and biochemical characteristics of SYL-3 strain.

Test index	Results (±)
Voges-Proskauer test	+
Citrate	–
Propionate	–
D-Xylose	+
L-Arabinose	+
D-Mannitol	+
Gelatin liquefaction	+
7%NaCl	+
PH5.7	+
Nitrate reduction	+
Starch hydrolysis	+

“+” Positive; “–” negative.

Aff irming the colonization ability of biocontrol strains on host plants would lay a foundation for using the strains for disease control in the field. The GPF fluorescence expressed from SYL-3-*gfp* can be observed by fluorescence microscopy, indicating that the strain was successfully transformed with the plasmid expressing GFP (Supplementary Figure 1A). By UV light irradiation, we observed that the SYL-3-*gfp* strain evenly covered the surface of tobacco leaves 2 days after the bacterial suspension was sprayed (Supplementary Figure 1B). Furthermore, the SYL-3-*gfp* was observed around tobacco leaf epidermal cells as well as in the vascular tissue on the fourth day after treatment (Supplementary Figures 1C,D). The results confirmed the good colonization ability of SYL-3 on tobacco leaves.

### SYL-3 exhibited a significant inhibitory effect against *A. alternata* and tobacco mosaic virus

**FIGURE 3:**
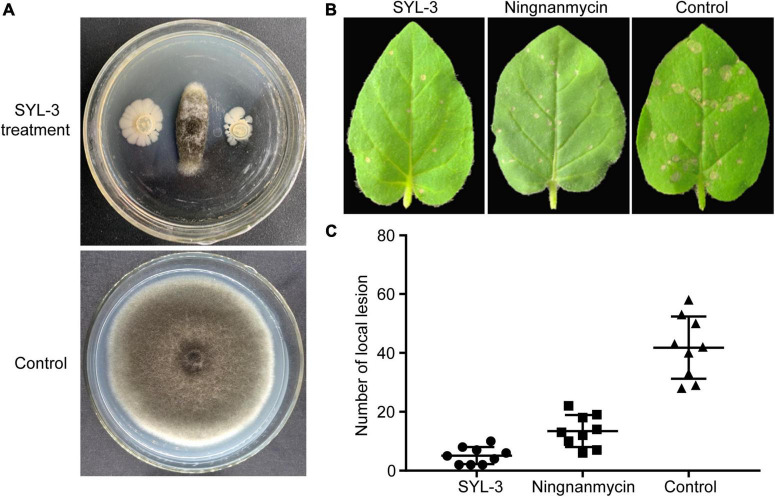
Antagonistic activities of SYL-3 against *A. alternata* and TMV. **(A)** Effects of SYL-3 on the mycelial growth of *A. alternata in vitro*. **(B)** Effects of SYL-3 or ningnanmycin on the necrotic lesions caused by TMV on *N. glutinosa* leaves. **(C)** Scatter plot of the necrotic lesions on *N. glutinosa* leaves treated with SYL-3, ningnanmycin, and sterile water (control).

To clarify the inhibitory effect of SYL-3 on *A. alternata*, we conducted an antibacterial test using *in vitro* confrontation method. The results showed that SYL-3 significantly reduced the mycelium growth of *A. alternata* by 71.33% ± 3.22 (Figure 3A). To clarify the effect of SYL-3 on the infection of TMV in plants, we assessed the inhibitory activity of SYL-3 bacterial suspension against TMV by investigating the number of necrotic spots on the inoculated leaves. It showed that SYL-3 reduced the necrotic spots by 87.77% ± 2.81 compared with the control treatment, suggesting a strong anti-TMV effect (Figures 3B,C).

**FIGURE 4:**
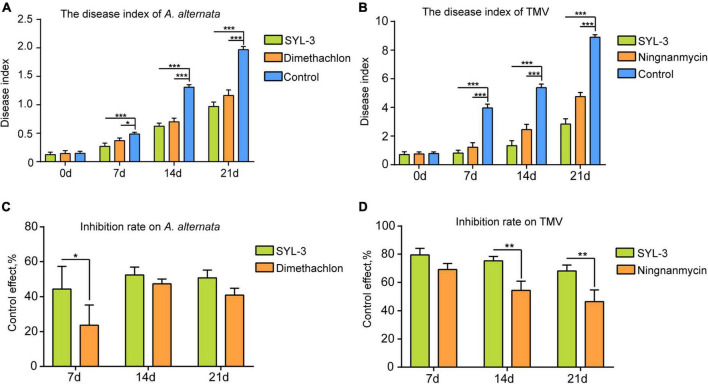
Plant disease index of *A. alternata* and TMV and control effect of SYL-3. **(A, B)** The disease index of *A. alternata* and TMV treated with SYL-3 on different days. **(C, D)** Inhibition rate on *A. alternata* and TMV treated with SYL-3 on different days (*p < 0.05, ** *p* < 0.01, and *** *p* < 0.001).

The field plot experiments were conducted in the selected tobacco planting field with perennially occurring foliar disease caused by *A. alternata* or TMV. We thereafter used the disease index of tobacco leaves to determine the inhibitory effect of SYL-3 on these diseases. The results showed that SYL-3 significantly suppressed the disease progression of *A. alternata* and reduced the respective disease index from 1.96 (control treatment) to 0.66 on day 21 after treatment (Figure 4A). Meanwhile, the disease index of *A. alternata* after dimethachlon treatment was 0.91. In addition, SYL-3 treatment also markedly suppressed mosaic leaf symptoms caused by TMV and lowered the disease index from 8.89 (control treatment) to 2.83 (Figure 4B). In contrast, the disease index after ningnanmycin treatment was down to 4.78. The inhibitory rates of SYL-3 against *A. alternata* and TMV were determined as 50.72% ± 3.63 and 68.11% ± 0.14, respectively, (Figures 4C,D), which was comparatively higher than that of dimethachlon or ningnanmycin. These results collectively indicated an effective inhibitory effect of SYL-3 against *A. alternata* and TMV *in planta* as well as in the field.

### Analysis of 16S rRNA and ITS1 sequencing data

Currently, many studies on microbial diversity are mainly based on the conserved region of nucleic acid sequences that encode ribosomal RNA as well as the ITS region (Zhuang et al., 2020). In this study, results of high-throughput sequencing analysis showed that 43,370 and 55,459 CCS were collected for SYL-3 and control treatment, respectively. A total of 38,097 and 53,599 high-quality optimization-CCS for bacteria were obtained after filtering the low-quality reads, chimeras, and attachment sequences, accounting for 88% and 97% of the total reads the number of these two treatments, respectively. Furthermore, there were 17,995 and 23,119 optimization-CCS obtained from SYL-3 and control treatment groups through ITS sequencing analysis, which accounted for about 96% and 99% of the total CCS (Table 2). The amount of sequencing data indicated that it was suff icient to reflect the species diversity in these samples.

**TABLE 2:** Statistics of sample sequencing data processing result.

Treatment		16s rRNA			ITS	
		
	Barcode-CCS	Optimization-CCS	Effective%	Barcode-CCS	Optimization-CCS	Effective%
SYL-3	43370 ± 860.97	38097 ± 969.32	87.84 ± 3.76	18838 ± 1375.95	17995 ± 1247.74	95.53 ± 1.14
Control	55459 ± 2342.28	53599 ± 2342.65	96.65 ± 0.50	23442 ± 264.52	23119 ± 201.80	98.62 ± 0.77

### Phyllosphere microbial community structure affected by SYL-3

**FIGURE 5:**
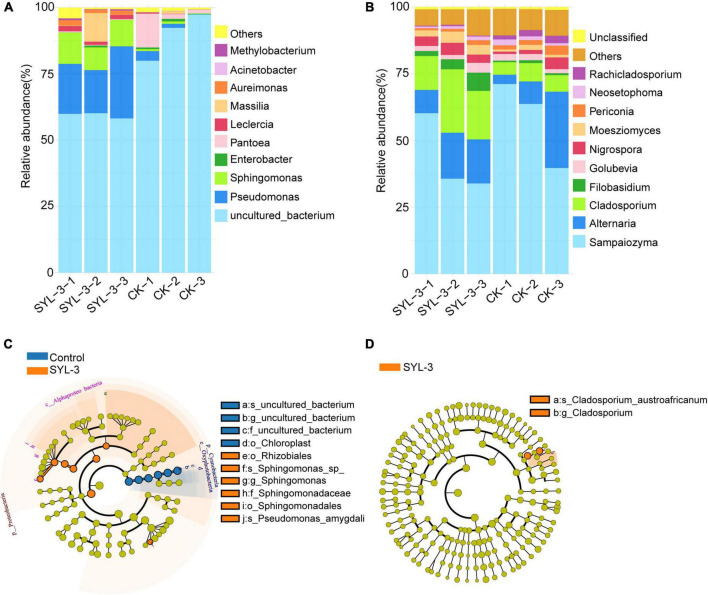
Comparative analysis of the phyllosphere dominant microbial taxa in samples collected from SYL-3 and the control treatment group. Relative abundance of several dominant bacterial genera **(A)** and fungal genera **(B)**. Comparison of microbial variations based on the LEfSe analysis of bacterial community **(C)** and fungal community **(D)** in the SYL-3 treatment and control treatment. Differences are represented by the color of the taxa (orange represents the SYL-3 treatment, blue represents the control treatment, and yellow represents the species with no significant difference).

The changes in the structure and composition of the phyllosphere microbial community are closely correlated with the impact of the external environment on the host plant (Vogel et al., 2020). At the genus level, we analyzed the phyllosphere microbial community structure in the control and SYL-3-treated groups on day 21 after treatment. The results showed the top 10 abundant kinds of the bacterial and fungal genus and revealed the effect of SYL-3 treatment on the phyllosphere microbial community structure of tobacco (Figures 5A,B). The abundances of *Pseudomonas*, *Sphingomonas*, and *Massilia* in the SYL-3 treatment group were increased by 19.00, 9.49, and 3.34%, respectively, compared with that of the control group (Supplementary Table 1), becoming the dominant genus of the bacterial community (Figure 5A). In contrast, the relative abundance of *Pantoea* decreased by 4.99% after SYL-3 treatment compared with the control group (Supplementary Table 1). The results of LEfSe analysis also indicated that *Pseudomonas* and *Sphingomonas* abundance were significantly increased compared with the control group (Figure 5C). Meanwhile, SYL-3 also affected the community structure of phyllosphere fungi, among which the abundance level of *Cladosporium* was markedly increased by 12.29% compared with the control group (Figures 5B,D and Supplementary Table 1). The abundance value of *Filobasidium* was also increased by 3.38%. On the contrary, the dominant fungus species *Sampaiozyma* decreased by 14.91% after SYL-3 treatment (Supplementary Table 1).

### Phyllosphere microbial community diversity affected by SYL-3

**FIGURE 6:**
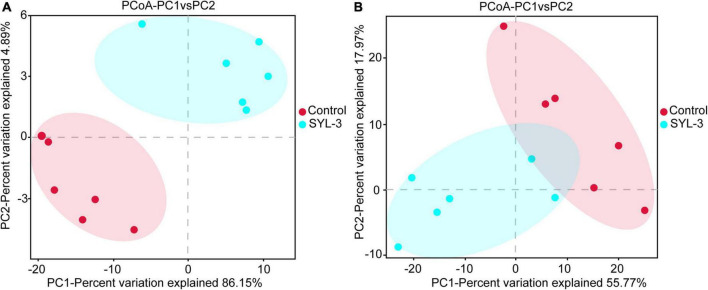
Taxonomic beta-diversity of phyllosphere microbial community as indicated by principal coordinates analysis (PCoA) plots. **(A)** PCoA analysis of bacterial community. **(B)** PCoA analysis of fungal community.

A rarefaction curve can be used to reflect the sequencing amount of microbiome samples (Wang et al., 2012). Here, the rarefaction curve of 16S rRNA and ITS1 sequencing gradually flattens out, indicating that the amount of sequencing data was suff icient to reflect the species diversity in samples (Supplementary Figure 2). In addition, to further elucidate the effects of SYL-3 on the diversity of tobacco phyllosphere microbial communities, we first analyzed the microbial community by using the diversity index. The results showed that the bacteria species richness (Chao1 and Ace index measure) and species diversity (Shannon index; Grice et al., 2009) of tobacco phyllosphere were significantly increased after SYL-3 treatment (Table 3). In contrast, the SYL-3 treatment resulted in a decrease in the Chao1 index of fungal diversity compared with that of the control treatment (Table 3). A principal coordinate analysis based on the Bray–Curtis algorithm was performed on phyllosphere microbial community samples. For fungal and bacterial communities, the spots representing the SYL-3 (red) or control (blue) treatment samples were separated (Figure 6A). In the total variance of the data set, the first two major components together comprised 94.93% and 86.77% of the total bacterial and fungal communities, respectively. In addition, the first principal component (PC1) was the most important, accounting for 86.15% and 55.77% of the total variation of bacterial (Figure 6A) and fungal communities (Figure 6B), respectively. These results revealed that phyllosphere microbial diversity significantly changed after SYL-3 treatment.

**TABLE 3:** Statistics of alpha diversity index of phyllosphere microbial community.

Treatment	Community characteristics					
	
	Bacterial community			Fungal community		
		
	ACE	Chao1	Shannon	ACE	Chao1	Shannon
SYL-3	65.14 ± 3.00	65.47 ± 3.91	1.49 ± 0.13	147.26 ± 4.47	140.71 ± 4.44	2.05 ± 0.26
Control	59.22 ± 7.48	58.33 ± 8.81	0.60 ± 0.31	144.65 ± 22.66	140.94 ± 18.78	1.84 ± 0.22

### Correlation between phyllosphere microbial community structure and disease incidence

**FIGURE 7:**
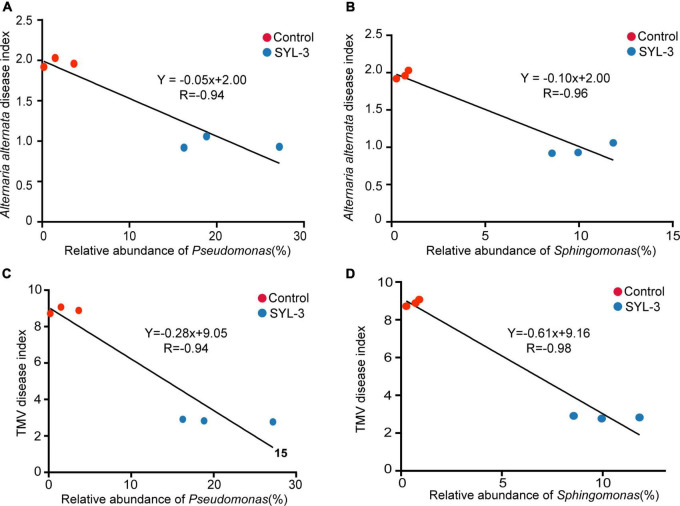
The correlation between the abundance of *Pseudomonas* and *Sphingomonas* and the disease index of *A. alternata* **(A, B)** and TMV **(C, D)**.

After 21 days of SYL-3 treatment, correlation analysis was performed between the abundance of dominant bacteria in tobacco phyllosphere and the disease index of common diseases. The results showed that the increase in the abundance of *Pseudomonas* and *Sphingomonas* in the phyllosphere microbes was negatively correlated (*P* < 0.05) with the disease index caused by *A. alternata* and TMV after SYL-3 treatment (Figure 7). In addition, analysis of phyllosphere microbial correlation community network showed that the relative abundance of *Pseudomonas* and *Sphingomonas* was positively correlated with the abundance of beneficial bacteria such as *Stenotrophomonas* and *Methylobacterium* (Supplementary Figure 3). Such correlation results suggested that changes in the population structure of *Pseudomonas* and *Sphingomonas* may indirectly affect the occurrence of plant diseases by SYL-3 treatment.

### Function of tobacco phyllosphere microbial community affected by SYL-3

**FIGURE 8:**
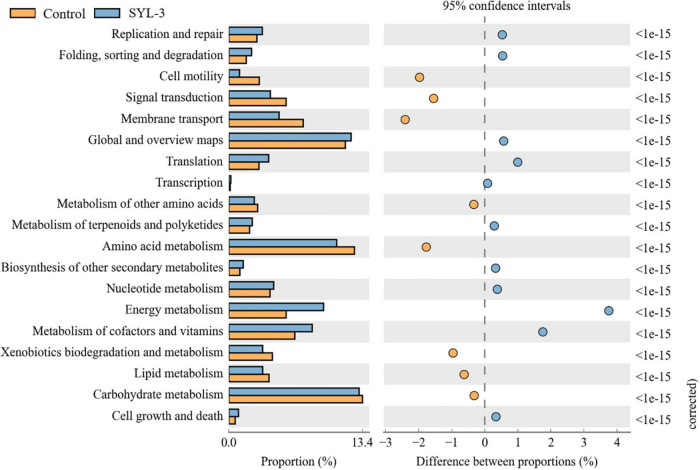
Metagenomes predicted by PICRUSt show significant differences in the functionality between SYL-3 treatment samples and controls.

PICRUSt software was used to predict the composition of functional genes in the samples by comparing the species composition information obtained from the 16S sequencing data of phyllosphere microorganisms. Then, the differences in metabolic pathways of functional genes in microbial communities between SYL-3 and control-treated samples were analyzed by KEGG (Figure 8). The KEGG metabolic pathways such as gene replication and repair, translation, nucleotide metabolism, energy metabolism, vitamin and cofactor metabolism, and the biosynthesis of other secondary metabolites were enriched after SYL-3 treatment compared with that of the control treatment. In contrast, the enrichment of pathways such as cell motility, membrane transport, and signal transduction decreased after SYL-3 treatment.

## Discussion

Understanding the structure and dynamics of microbial communities is of great significance due to their effects on plant health (Gobbi et al., 2020). Previous investigations in biological control have mainly focused on soil microbial communities and revealed their critical roles in crop disease management (Palmieri et al., 2016; Shi et al., 2016, 2017). As many foliar pathogens colonize on leaf surfaces before infection, the regulation of the phyllosphere microbial community has become a new subject in biological control (Vorholt, 2012). In this study, the potential relationship among the newly isolated beneficial *B. velezensis* strain SYL-3, the phyllosphere microbial community of tobacco, and the occurrence of two ubiquitous plant diseases was investigated using microbiome high-throughput sequencing approaches.

Among the reported beneficial microorganisms and biological agents, *Bacillus* spp. has been widely applied in the management of various plant diseases (Shafi et al., 2017; Yang et al., 2020; Zhou et al., 2021). Here, phylogenetic analysis indicated that SYL-3 showed high sequence identity and clustered closely with species *B. velezensis*. Previous investigations reported that *B. velezensis* produced a variety of metabolites, regulating the microbial community structure, and can effectively inhibit a variety of crop diseases. For instance, *B. velezensis* FZB42 eff iciently antagonizes *Phytophthora sojae* by producing bacilysin (Han et al., 2021). *B. velezensis* B-4 is effective in controlling *Sclerotinia sclerotiorum* by synthesizing non-ribosomal peptide synthetases, polyketide synthases, and lantipeptide synthesis proteins (Zhu et al., 2020). *B. velezensis* T052-76 can control sweet potato foot-rot disease caused by *Plenodomus destruens* by altering the structure of the indigenous bacterial community (Mateus et al., 2019).

Our results showed that SYL-3 significantly affected the relative abundance of foliar bacterial communities compared with those of the fungus. Especially, the genera of *Sphingomonas* and *Pseudomonas* increased observably and became the dominant bacterial genera under SYL-3 treatment. *Sphingomonas* sp. is a Gram-negative, rod-shaped aerobic bacterium that possesses multifaceted functions ranging from improving plant fitness to protecting plants from diseases (Innerebner et al., 2011; Asaf et al., 2020). The researchers demonstrated that the foliar bacterium *Sphingomonas* sp. could protect plants against the leaf-pathogenic *Pseudomonas syringae* through substrate competition (Vogel et al., 2012). The lesion coverage rate (LCR) of angular leaf spot of cucumber was correlated negatively with the abundance of *Sphingomonas* in the phyllosphere microbial community (Luo et al., 2019). *Sphingomonas* isolated from tomato leaves had strong *in vitro* antifungal activity against *B. solani*, one of the tomato pathogens (Enya et al., 2007). Herein, our research indicated that the abundance of *Sphingomonas* was negatively (*r* = –0.96) associated with *A. alternata* disease index, which was consistent with earlier reports. *Pseudomonas* sp. is a Gram-negative bacterium, which has been reported to inhibit plant diseases caused by *Ralstonia solanacearum*, *Phytophthora capsici*, and *Rhizoctonia solani* (Li, 2018; Shi, 2019), as well as induce host systemic resistance and improve morphological and biochemical traits of crops (Kumar Yadav et al., 2021). Here, the abundance of *Pseudomonas* was negatively correlated with the TMV disease index (*r* = –0.94; Figure 7C), which is also consistent with previous reports that the *Pseudomonas* can inhibit the incidence of TMV in tomato plants and induce plant resistance (Gupta et al., 2021). Additionally, the results showed that *B. velezensis* could be detected but not as the dominant strain in the phyllosphere (Supplementary Table 1). Nevertheless, based on the good colonization capability of SYL-3, we reasonably speculate that SYL-3 treatment can exhibit a sustainable regulatory effect on phyllosphere microbial communities as well as plant pathogens.

Changes in fungal communities in the treatment groups were not as dramatic as those in bacterial communities, with only a significant increase in the abundance of *Cladosporium*. Studies have shown that *Cladosporium* produced secondary metabolites like phenylacetic acid, p-hydroxyphenyl acetic acid, and p-hydroxyphenyl ethanol, exhibiting good antibacterial and antifungal activities (Hwang et al., 2001; Kim et al., 2004; Ding et al., 2008). It has been reported that the volatile organic compounds (VOCs) produced by *Cladosporium* could promote the growth and development of tobacco as well as improve its disease resistance (Paul and Park, 2013). Additionally, *Cladosporium* conidia can induce hypersensitive responses when in contact with tobacco leaves, activating defense responses in tobacco cells (Mattos et al., 2018). Likewise, metagenomic functional analysis of the PICRUSt predictions conducted in this study also confirmed that pathways for the biosynthesis of secondary metabolites were enriched in the treatment groups (Figure 8), which suggested that the decrease in the incidence of tobacco diseases in the treatment group may also be associated with the increase in the abundance of *Cladosporium*. Taken together, SYL-3 treatment induced the colonization of *Sphingomonas*, *Pseudomonas*, *Cladosporium*, etc. in foliar microorganisms, and then the beneficial microflora above contributed to inhibiting the occurrence and progression of diseases (Figures 5, 7). However, considering which microorganism was the one playing a leading role in the disease-inhibiting event, or whether it was the result of the synergistic effect of multiple microorganisms, we still need further research on the isolation and culture of phyllosphere microorganisms.

Microbial diversity was identified as a key factor in preventing diseases and can be implemented as a biomarker in plant protection strategies (Berg et al., 2017). Several studies indicated that a relationship has been found between microbial diversity and root disease suppression. For example, traditional crop rotation reduces the outbreak potential of pathogenic microorganisms by enhancing the overall microbial diversity in the soil (Mazzola, 2004). *B. amyloliquefaciens* FZB42, as a commercial and eff icient plant strengthener, can resist *R. solani* by enhancing the overall microbial diversity (Erlacher et al., 2014). *B. subtilis* Tpb55 can effectively inhibit *Phytophthora parasitica* var. *nicotianae* by increasing bacterial diversity in tobacco rhizosphere soil (You et al., 2014). In this study, the alpha diversity of the microbial community in the SYL-3 treatment group was significantly increased, and the beta diversity of the microbial community was significantly spatially differentiated. And through the microbial correlation network analysis, it was also found that due to the increase in the diversity of phyllosphere microbes, the correlation between microbes also increased, which may lead to the formation of new homeostasis between plants and phyllosphere microbes. This is consistent with the conclusion of previous studies that greater microbial diversity has a more beneficial impact on crop resistance to pathogens (Shi et al., 2017; Han et al., 2018).

Validating the function of the microbiome is the key to revealing their relationship with the environment (Escalas et al., 2019). Inferring functions based on the diversity of bacteria at present is diff icult since bacteria often transfer genes and exhibit a high degree of dependence and redundancy (Klindworth et al., 2013). Currently, PICRUSt analysis is widely explored to acquire functional insights into the microbial community (Langille et al., 2013). This study found that several pathways that are significantly enriched after treatment, such as cofactors and vitamin metabolism pathways, are related to the promotion of plant resistance by microbial treatment (Sun et al., 2021). Studies have shown that DNA damage repair pathways mainly determine genome integrity and plant survival (Raina et al., 2021). Meanwhile, the biosynthesis of secondary metabolites of microorganisms is recognized as a rich source of biomolecules with potential medicinal applications (Li and Tan, 2017). Therefore, SYL-3 application significantly affected the function of the phyllosphere microbiome. At the same time, it indicated that SYL-3 may indirectly increase host resistance through the induction of host critical genes or pathways from the transcriptional level of some common resistance indicator genes and content of active oxygen (data not shown). Thus, it also showed the potential application value of SYL-3 as a resistance inducer for a wider range of other plant disease control.

In this study, SYL-3 belonging to *B. velezensis* was isolated and identified with a robust inhibitory effect on the infection of *A. alternata* and TMV. SYL-3 increased the abundance of several beneficial microorganisms such as *Pseudomonas*, *Sphingomonas*, and *Massilia* as well as enhanced the overall diversity of the phyllosphere microorganisms, which largely contributed to the induction of resistance against *A. alternata* or TMV. This work will improve the understanding of action modes on biocontrol for SYL-3 and provide a potential sustainable management strategy for plant diseases caused by *A. alternata* and TMV.

## Data availability statement

The original contributions presented in this study are publicly available. This data can be found here: The raw reads generated by PacBio sequencing were submitted to the Sequence Read Archive database at NCBI (SRA; http://www.ncbi.nlm.nih.gov/Traces/sra), with the SRA BioProject accession numbers [PRJNA790673 (Bacterial communities 16s rRNA sequencing results)] and [PRJNA790671 (Fungal communities ITS sequencing results)].

## Author contributions

ZW, YW, and HL conceived and designed the experiments, carried out the transcriptome analysis, and analyzed the data. HL, JJ, MA, BL, and YX performed the main experiments. HL and JJ contributed equally. CX, LJ, and HL cultivated *N. benthamiana* plants and *N. tabacum* cv. NC89 and treated samples for transcriptome sequencing. HL, MA, FY, JJ, and ZW prepared reagents, materials, and analysis tools. ZW, JJ, and HL prepared the figures and tables. HL wrote the original draft. ZW, YW, BL, and CX reviewed drafts of the manuscript. All authors reviewed and approved the manuscript.

## Footnotes

1
http://drive5.com/usearch/manual/uchime_algo.htm

2
http://www.drive5.com/usearch/

3
https://sourceforge.net/projects/rdp-classifier/

4
http://www.mothur.org/

5
http://www.ncbi.nlm.nih.gov/Traces/sra

## Supplementary material

Supplementary Figure 1 Colonization of SYL-3-*gfp* strain on tobacco leaves. **(A)** Confocal microscope observation of SYL-3-*gfp* strain. **(B)** UV irradiation observation of the distribution of SYL-3-*gfp* strain on the leaf surface 2 days after spraying. **(C, D)** Confocal microscopy observation of the colonization of SYL-3-*gfp* in leaf epidermal cells and vascular tissue.

Supplementary Figure 2 Sample rarefaction curve. **(A)** Rarefaction curve for bacteria in samples. **(B)** Rarefaction curve for fungus in samples.

Supplementary Figure 3 Pearson’s correlation network analyses at genus level of phyllosphere microbial communities in SYL-3 treatment. Circles represent species, the size of the circle represents the abundance, the edges represent the correlation between the two species, the thickness of the edge represents the strength of the correlation and the color of the line: orange represents the positive correlation and green represents the negative correlation.
