# Supplementary figures and images for: Bacillus velezensis SYL-3 suppresses Alternaria alternata and tobacco mosaic virus infecting Nicotiana tabacum by regulating the phyllosphere microbial community
Source: Front Microbiol. 2022 Jul 28;13:840318. doi: 10.3389/fmicb.2022.840318 (PMC9366745; doi:10.3389/fmicb.2022.840318)

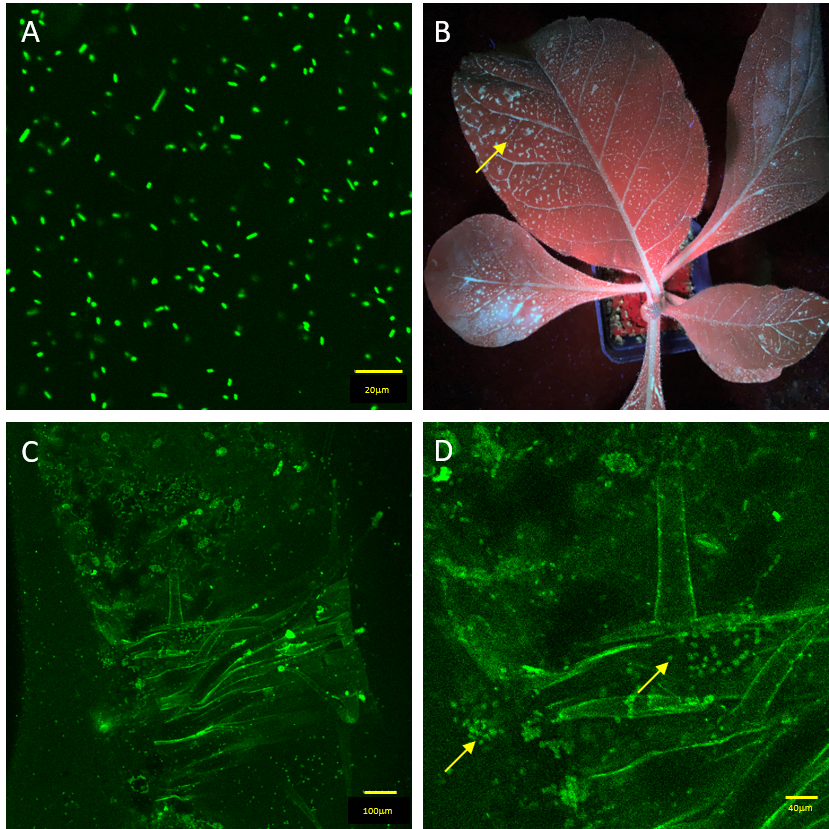

Supplement: Supplementary Figure 1 — Colonization of SYL-3-gfp strain on tobacco leaves. (A) Confocal microscope observation of SYL-3-gfp strain. (B) UV irradiation observation of the distribution of SYL-3-gfp strain on the leaf surface 2 days after spraying. (C, D) Confocal microscopy observation of the colonization of SYL-3-gfp in leaf epidermal cells and vascular tissue. [file Image_1.tif]

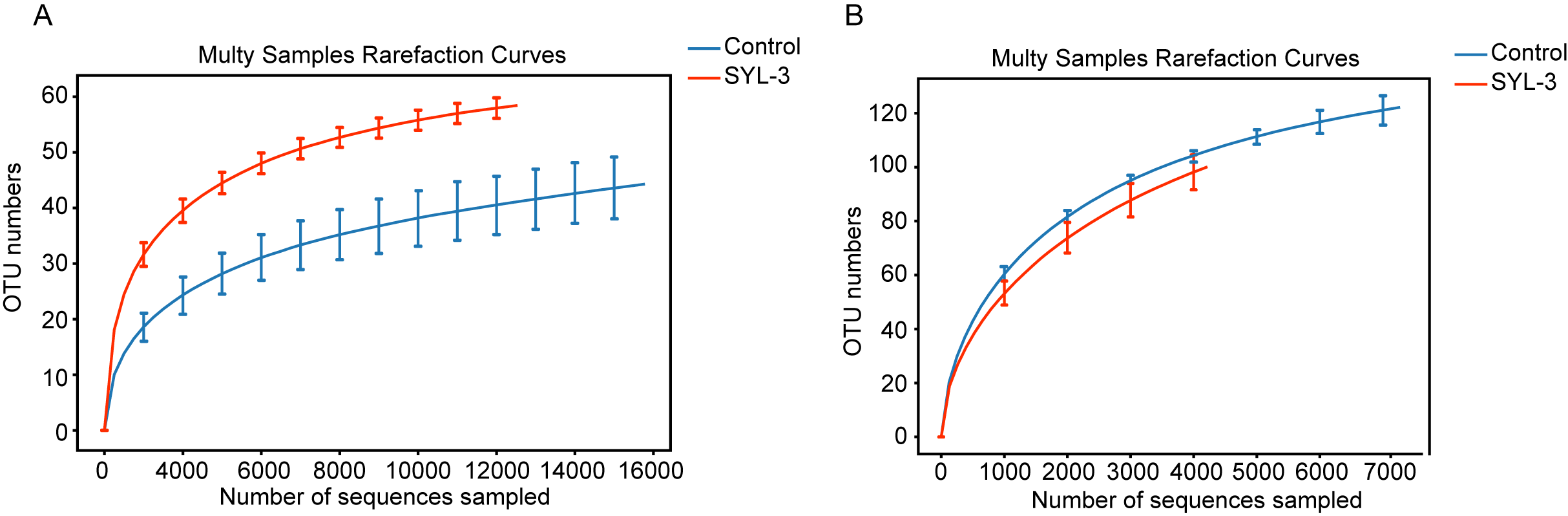

Supplement: Supplementary Figure 2 — Sample rarefaction curve. (A) Rarefaction curve for bacteria in samples. (B) Rarefaction curve for fungus in samples. [file Image_2.tif]

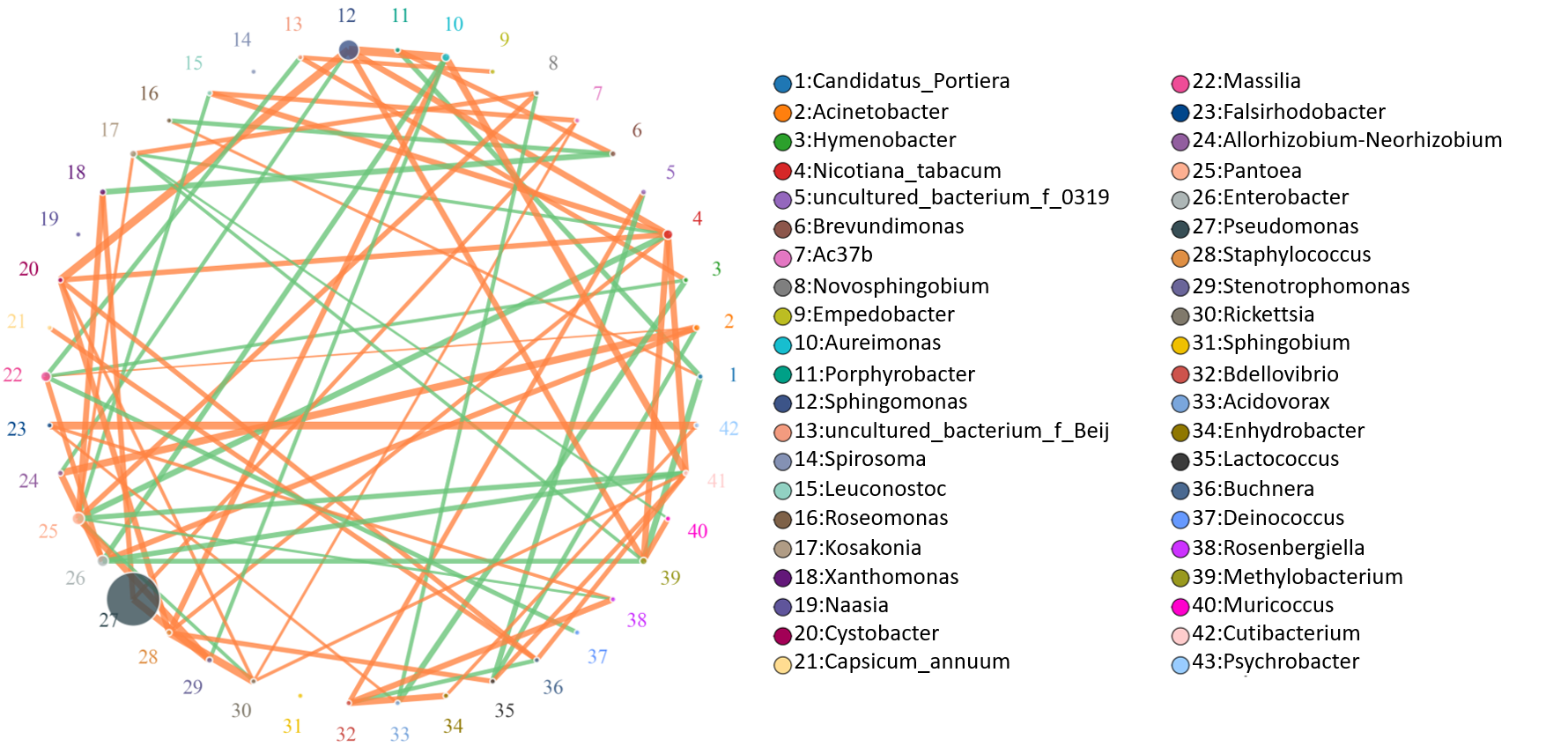

Supplement: Supplementary Figure 3 — Pearson’s correlation network analyses at genus level of phyllosphere microbial communities in SYL-3 treatment. Circles represent species, the size of the circle represents the abundance, the edges represent the correlation between the two species, the thickness of the edge represents the strength of the correlation and the color of the line: orange represents the positive correlation and green represents the negative correlation. [file Image_3.tif]
